# Clinical Outcomes with Prospective Brain Sensing Data Following Bilateral Globus Pallidus Deep Brain Stimulation in X‐Linked Dystonia Parkinsonism

**DOI:** 10.1002/mdc3.70044

**Published:** 2025-03-15

**Authors:** Eoghan Donlon, Clodagh O'Keeffe, Jack Horan, Frederica Ruggieri, Jessica Fitzpatrick, Michael O'Neill, Michael Alexander, Conor Fearon, Catherine Moran, Richard A. Walsh

**Affiliations:** ^1^ Dublin Neurological Institute Mater Misericordiae University Hospital Dublin Ireland; ^2^ School of Medicine University College Dublin Dublin Ireland; ^3^ Department of Neurosurgery Beaumont Hospital Dublin Ireland; ^4^ Department of Radiology Beaumont Hospital Dublin Ireland; ^5^ Department of Neurophysiology Tallaght University Hospital Dublin Ireland

**Keywords:** DYT3 dystonia, Lubag, deep brain stimulation, local field potentials

Deep brain stimulation (DBS) of the globus pallidus interna (GPi) is effective in providing symptomatic relief of dystonia in X‐linked dystonia‐parkinsonism (XDP or DYT3/PARK TAF1), whereas later evolving parkinsonism appears to be stimulation and drug refractory.^1^, ^2^, ^3^ One report demonstrates an acute suppression of potentially pathogenic beta frequency hypersynchrony in XDP.^4^ No data exists on prospective sensing data in this patient cohort beyond the acute period following implantation. Here we describe local field potential (LFP) characteristics in two cases of early bilateral GPi‐DBS in XDP.^3^

Patient 1: A 32 year‐old Filipino male presented with a 6‐month history of lower limb pain, stiffness and weight loss. He complained of spasms of abdomen and hip flexors, involuntary lower limb elevation, only improved by walking. On examination, he was cachectic with a flexed posture, visible abdominal contractions and mild hypomimia. Stride length was reduced with multi‐point turning (Video 1). Testing confirmed a TAF1 mutation consistent with XDP. Despite pharmacotherapy, his condition deteriorated rapidly necessitating nasogastric feeding. A decision was made to move to early bilateral GPi‐DBS (Percept PC device, Medtronic Inc.) 12 months after his first presentation (Fig. S1).

**Video 1:** Clinical examination of patient 1 pre and post deep brain stimulation (DBS) insertion and activation.

Following DBS activation and optimization (Table S1) there was improvement in all dystonic symptoms with improvement in BFMDRS (17–6.5, 62%) and the X‐linked Dystonia Parkinsonism Rating Scale (XDPRS) (49–16, 68%) at 1 year post‐operatively. The patient now has minimal disability, no pain or dystonic spasms and has returned to fulltime work. Analysis of local field potentials with using the BrainSense™ survey was performed at baseline and subsequent assessments. LFP analysis identified two peaks of interest, both in the beta range (15–18 hz and 25–30 hz) in both hemispheres. Over follow‐up there was consistent suppression of these two peaks at 1, 3, 6 and 18 months, which correlated with observed clinical improvement in BFMDRS and XDP‐MDSPRS (Fig. 1).

**Figure 1:**
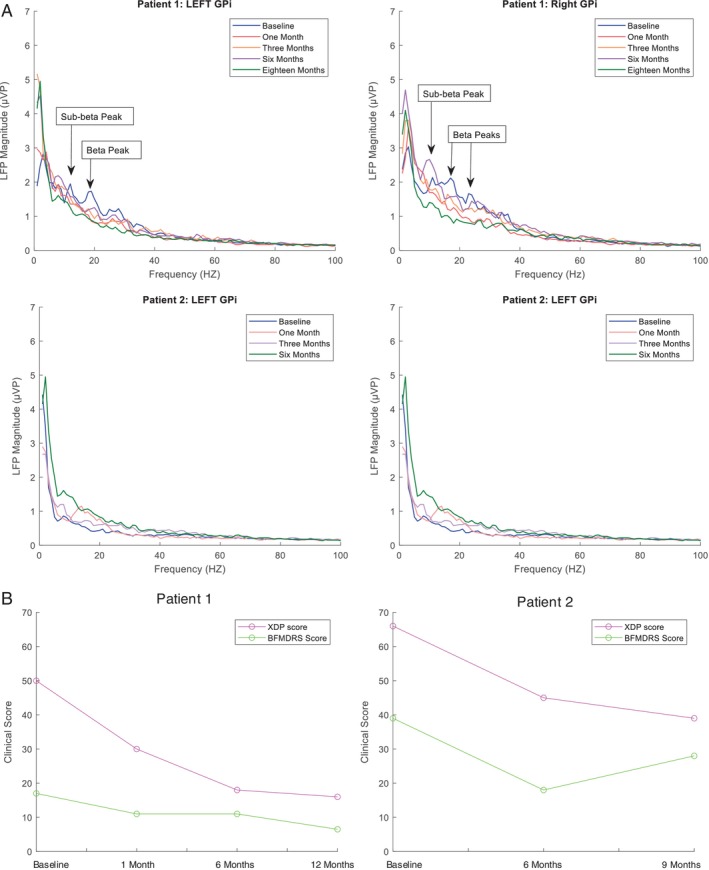
(A) Local field potential (LFPs, expressed as μVP/Hz) recordings from each hemisphere at baseline and subsequent recordings for both patients. Patient 1: Labeled LFP peaks of interest left globus pallidus interna (GPi)‐18 Hz (Beta) and 12 Hz (Sub‐Beta), Right GPi 17 and 23 Hz (Beta) and 11 Hz (Sub‐Beta). Patient 2: no LFP peaks of interest. (B) Clinical scales; X‐linked dystonia parkinsonism rating scale (XDPRS) and Burke Fahn Marsden Dystonia Rating scale (BFMDRS) measured at baseline, 1, 6, and 12 months for patient 1 and baseline, 6 and 9 months for patient 2.

Patient 2: A 47 year‐old Filipino male presented with a 6 month history of progressive dysarthria, dysphagia and weight loss. On examination, there was evident hypomimia and prominent dysarthria. There was cervical dystonia with prominent orolingual dystonia and continuous phasic tongue contractions evident with the mouth open. There was increased axial tone and stooped gait (Video 2). Genetic testing identified a TAF1 mutation. There was no symptomatic response to levodopa or amantadine. With ongoing difficulty with communication and swallow, a decision was made to move to early GPi‐DBS (Fig. S1).

**Video 2:** Clinical examination of patient 2 pre and post deep brain stimulation (DBS) insertion and activation.

There was moderate improvement in BFMDRS (39–28, 28%) and XDP‐MDSPRS (66–39, 41%) at 9 months. There was no evident improvement in the lingual dystonia and associated dysarthria over the post‐operative period, however, turning off stimulation to run a BrainSense survey resulted in the onset of severe intolerable lingual dystonia and he was not willing to have a survey run routinely at follow‐up. No LFP frequency bands of interest were identified or suppression was seen on repeat surveys (Fig. 1).

Elevated power in lower sub‐beta frequency ranges have been consistently identified as possible correlates of basal ganglia‐thalamo‐cortical dysfunction in all forms of dystonia.^5^ Reduction in the 4–12hz LFP band in cervical dystonia in response to high frequency GPi stimulation supports low‐frequency pathologic oscillations as a putative biomarker if not pathological driver underlying dystonic contractions.^6^ In the single report of LFP characteristics in XDP, Whitmer and colleagues describe a clear beta range (20–30 hz) LFP peaks in the GPi in their patient, which attenuated with stimulation.^4^ The first case echoes this finding, but in keeping with most of the literature an additional sub‐beta peak was identified and for the first time adds the observation that sustained and progressive suppression of beta range hypersynchrony correlates with clinical improvement.

We were unable to identify a clear peak in beta or lower frequencies in our second case. This was a different phenotype with focal bulbar dystonia predominating and no axial or appendicular dystonia. It is known that craniofacial dystonia tends to more refractory to GPi‐DBS with respect to cervical and truncal dystonia.^7^ There was a striking deterioration after turning off stimulation that suggests some useful benefit from surgery that may have had a role in preventing a deterioration. Primary craniocervical dystonia is less well studied with respect to LFPs. One series of 28 patients with Meige's syndrome by Zhang and colleagues identified a theta frequency biomarker for dystonia relating to the duration of the theta burst frequency rather than augmented spectral power.^8^ Our second case with no clear peak in LFP spectral power in either beta or sub‐beta frequencies may be consistent with this finding in other bulbar predominant cases (Data S1).

On the basis of a single XDP patient with identifiable twin beta and theta range peaks, we are unable to say which of the observed spikes in frequency power are linked to the dystonic or parkinsonian element of the disorder. The paucity of parkinsonian signs in patient 1, the gratifying response of dystonia (89% improvement in XDP‐MDSP dystonia subscale) to stimulation and the relatively modest improvements in parkinsonian signs (43% in parkinsonism subscale) may support the beta suppression as the correlate for dystonia. Larger collective data sets are needed to clarify if beta range hypersynchronisation is a marker of dystonia and potential biomarker of therapeutic response in XDP.

## Author Roles

(1) Research project: A. Conception, B. Organization, C. Execution; (2) Statistical Analysis: A. Design, B. Execution, C. Review and Critique; (3) Manuscript Preparation: A. Writing of the first draft, B. Review and Critique.

E.D.: 1A, 1B, 2A, 2B, 2C, 3A, 3B

C.O.K.: 1B, 2A, 2B, 2C, 3B

J.H.: 1C, 2C, 3C

F.R.: 2B, 2C

J.F.: 2A, 2B

M.O.N.: 2A, 2B

C.F.: 2C, 3B

C.M.: 2C, 3B

R.W.: 1A, 1B, 2C, 3B

## Disclosures

**Ethical Compliance Statement:** The authors confirm that the approval of an institutional review board was not required for this work. We confirm that we have read the Journal's position on issues involved in ethical publication and affirm that this work is consistent with those guidelines.

**Funding Sources and Conflicts of Interest:** No specific funding was received for conducting this study. ED received salary funding support from Boston Scientific, the other authors have no conflicts of interest to declare.

**Financial Disclosures for the Previous 12 Months:** The authors declare that there are no additional disclosures to report.

## Patient Consent for Publication

Consent for publication was gained from the patients.

## Supporting information


**Data S1.** Reviewer comments and responses from authors.

**Figure S1.** Brainlab stereotactic planning images of MRI trajectory of leads post‐operatively. Patient 2 right deep brain stimulation (DBS) lead marginally deeper placement, otherwise all leads well positioned within the globus pallidus interna (GPi).

**Table S1.** Summary of programming iterations from baseline to each subsequent visit for both patients.

## Data Availability

The data that support the findings of this study are available from the corresponding author upon reasonable request.
