# Supplementary figures and images for: Clinical Outcomes with Prospective Brain Sensing Data Following Bilateral Globus Pallidus Deep Brain Stimulation in X‐Linked Dystonia Parkinsonism
Source: Mov Disord Clin Pract. 2025 Mar 15;12(7):1024–7. doi: 10.1002/mdc3.70044 (PMC12275003; doi:10.1002/mdc3.70044)

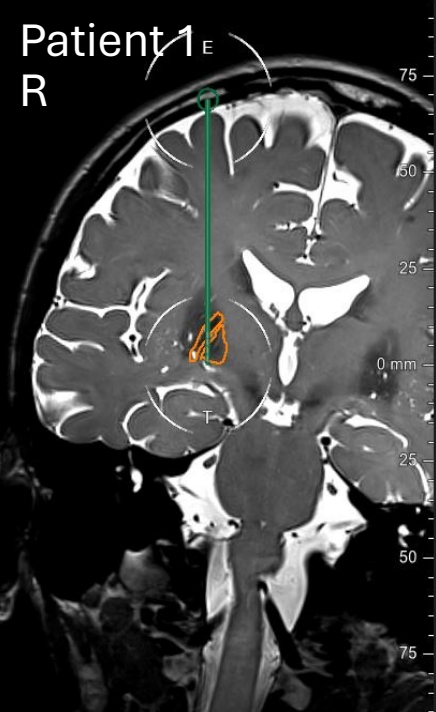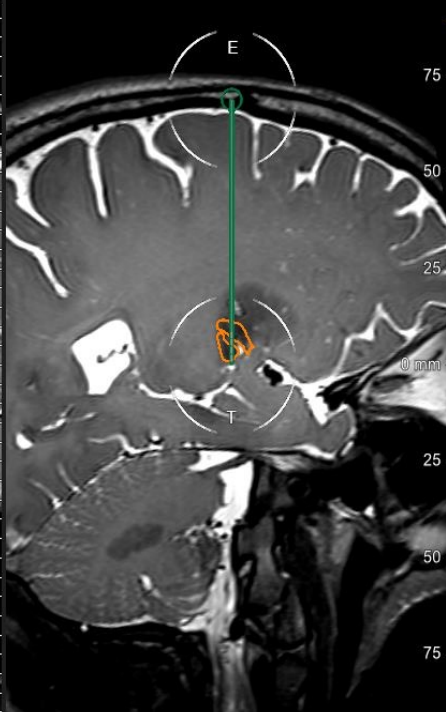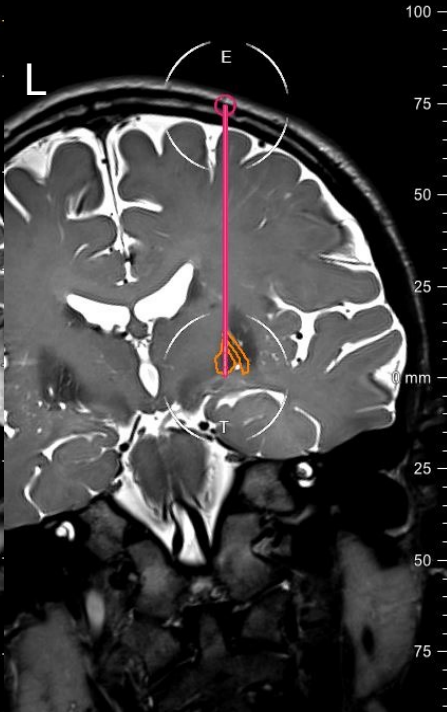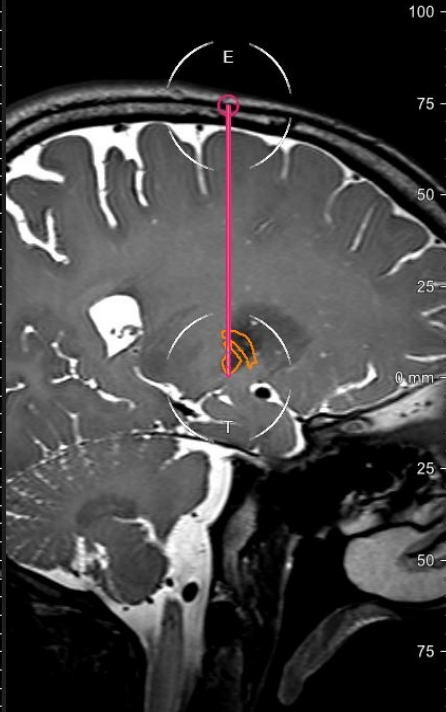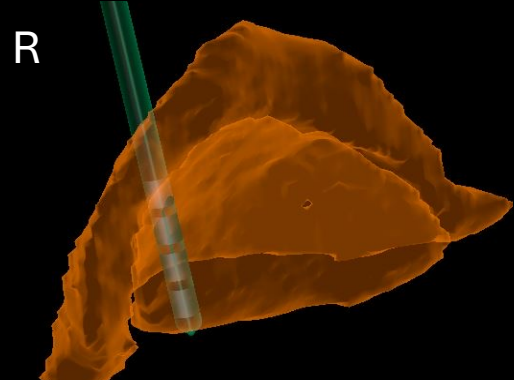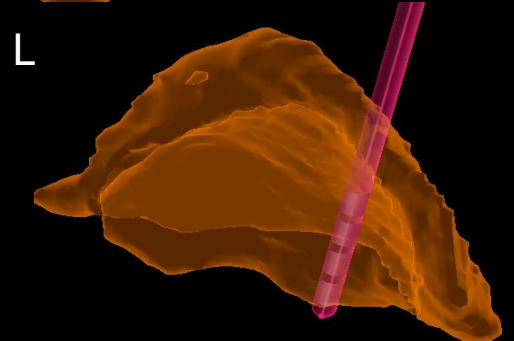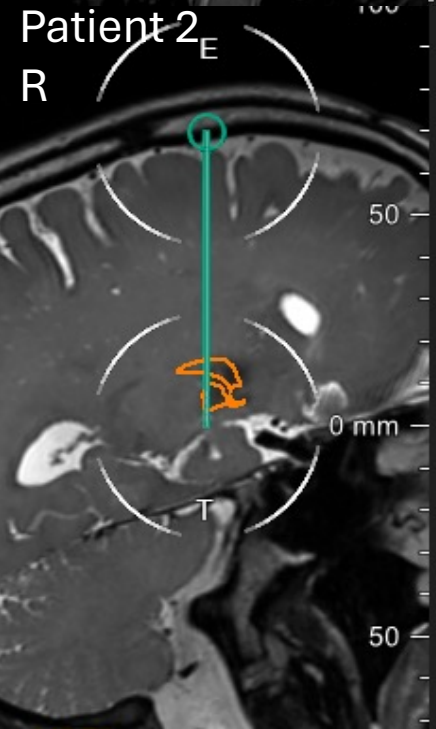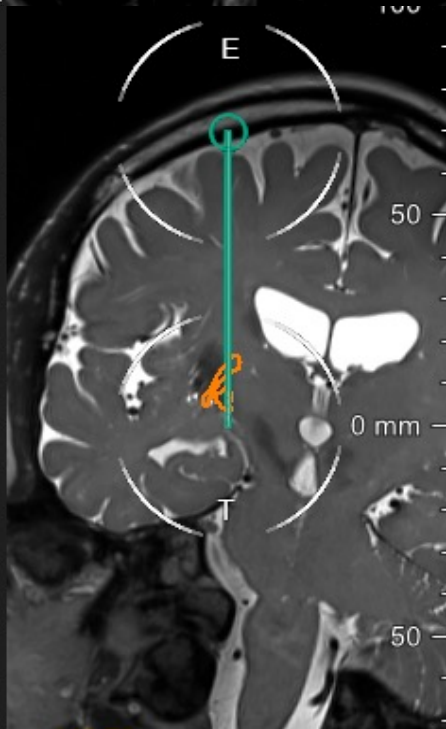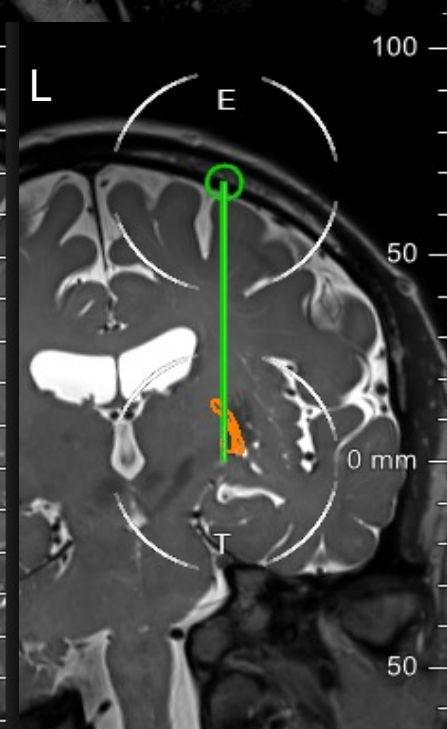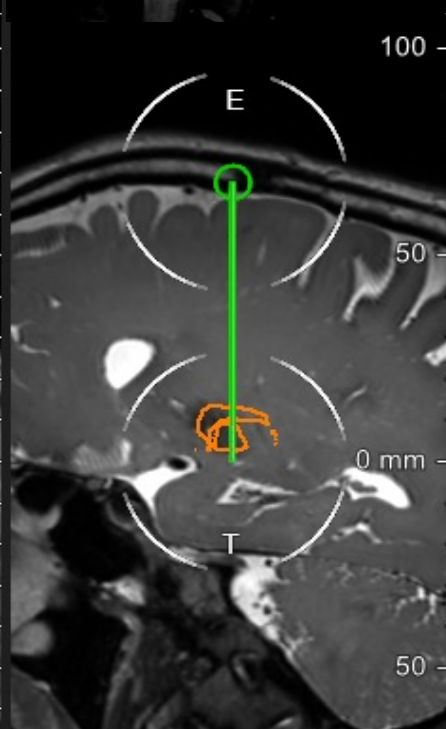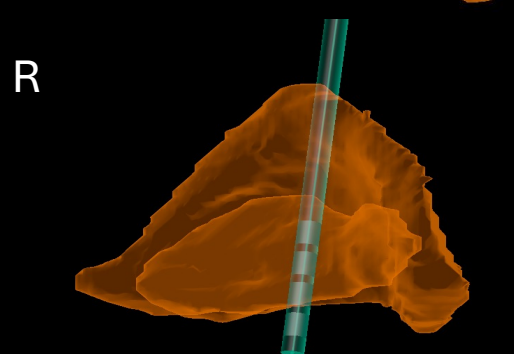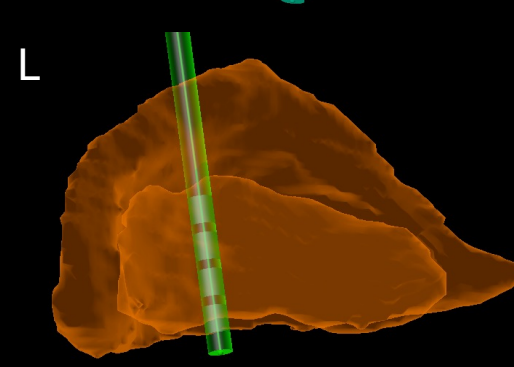

Supplement: Supplementary file 2 — Figure S1. Brainlab stereotactic planning images of MRI trajectory of leads post‐operatively. Patient 2 right deep brain stimulation (DBS) lead marginally deeper placement, otherwise all leads well positioned within the globus pallidus interna (GPi). [file MDC3-12-1024-s001.pdf]
