# Supplementary material for: Clinical Outcomes with Prospective Brain Sensing Data Following Bilateral Globus Pallidus Deep Brain Stimulation in X‐Linked Dystonia Parkinsonism
Source: Mov Disord Clin Pract. 2025 Mar 15;12(7):1024–7. doi: 10.1002/mdc3.70044 (PMC12275003; doi:10.1002/mdc3.70044)
Supplement: Supplementary file 3 — Table S1. Summary of programming iterations from baseline to each subsequent visit for both patients. [file MDC3-12-1024-s003.docx]

**Supplementary Table 1: Programming summary**

Patient 1

| Visit schedule | Left GPi | Right GPi | Clinical assessment/changes |
| --- | --- | --- | --- |
| BL | 1- (a, b, c) PW: 60 125hz 2.5mA | 9- (a, b,c) PW: 60 125hz 2.5mA | - Level 2 best contact on sides. Acute reduction of bilateral lower limb dystonia. - Dysarthria limiting S/E b/l. |
| 3M | Entry settings  1- (a, b, c) PW: 60 125hz 3.4mA  Exit settings  1- (a, b, c) PW: 60 125hz 3.5mA | Entry settings  9- (a, b, c) PW: 60 125hz 3.0mA  Exit settings  9- (a, b) PW: 60 125hz 9a: 1.2mA, 9b: 1.2mA | - STIM increased after discussion over the phone to address return of lower limb dystonia. - Lower limb/truncal dystonia moderately improved. - New blepharospasm. - Mild dysarthria, driven predominantly by right GPi - Directional steering right GPi 9- rt reduced dysarthria significantly (a + b) 3.8mA total i.e 9c driving medial dysarthria. |
| 6M | Entry settings  1- (a, b, c) PW: 60 125hz 3.5mA  Exit settings  1- (a, b, c) PW: 60 125hz 3.5mA | Entry settings  9- (a, b) PW: 60 125hz 9a: 1.2mA, 9b: 1.2mA  Exit settings  9- (a, b) PW: 60 125hz 9a: 1.0mA, 9b: 1.0mA | - Lower limb/truncal dystonia significantly improved. - Blepharospasm improved. - Mild dysarthria, improved. with reduced rt GPi STIM. - Returned to work. |
| 9M | Entry settings  1- (a, b, c) PW: 60 125hz 3.4mA  Exit settings  1- (a, b, c) PW: 90 125hz 3.6mA | Entry Settings  9- (a, b) PW: 60 125hz 9a: 1.0mA, 9b: 1.0mA  Exit settings  9- (a, b) PW: 60 125hz 9a: 1.0mA, 9b: 1.0mA | - Botox improved Blepharospasm (70%). - Some return of rt leg dystonia lying flat. - Increased PW to 90 in Lt GPi. |
| 12M | Entry settings  1- (a, b, c) PW: 90 125hz 3.6mA  Exit settings  1- (a, b, c) PW: 90 125hz 3.6mA | Entry settings  9- (a, b) PW: 60 125hz 9a: 1.0mA, 9b: 1.0mA  Exit settings  9- (a, b) PW: 60 125hz 9a: 1.0mA, 9b: 1.0mA | - Speech stable. - No axial or limb dystonia - Minimal blepharospasm. - STIM unchanged. |
| 15M | Entry settings  1- (a, b, c) PW: 90 125hz 3.6mA  Exit settings  1- (a, b, c) PW: 60 125hz 3.7mA | Entry settings  9- (a, b) PW: 60 125hz 9a: 1.0mA, 9b: 1.0mA  Exit settings  9- (a, b) PW: 50 125hz 9a: 1.0mA, 9b: 1.0mA | - Speech deteriorated. - Both sides driving dysarthria L > R. - L2 ring mode Rt GPi speech much worse, 9c clearly driving some speech dysfunction. Reduced rt STIM immediate return of pulling in trunk. - Reduced PW left and right GPi improved speech. |
| 18M | Entry settings  1- (a, b, c) PW: 60 125hz 3.7mA  Exit settings  1- (a, b, c) PW: 60 125hz 3.7mA | Entry settings  9- (a, b) PW: 50 F: 125hz 2.8mA (a: 1.0mA, b: 1.0mA)  Exit settings  9- (a, b) PW: 50 125hz 9a: 1.0mA, 9b: 1.0mA | - Not aware of dystonia. - Speech improved significantly. - Minimal blepharospasm. |

Patient 2

| Visit schedule | Left GPi | Right GPi | Clinical assessment/changes |
| --- | --- | --- | --- |
| BL | Group A  1- (a, b, c) PW: 60 125hz 3.5mA  Group B  0- (a, b, c) PW: 60 125hz 3.0mA | Group A  9- (a, b, c) PW: 60 125hz 3.5mA  Group B  8- (a, b, c) PW: 60 125hz 3.5mA | - Most ventral contacts (level 1 and 2) best anatomically positioned and best therapeutic windows. - Small therapeutic windows in upper contact pairs - Trial 1 month each group. |
| 3/12 | Entry settings  Group B  0- (a, b, c) PW: 60 125hz 3.0mA  Exit settings  Group A  0- (a, b, c) PW: 60 125hz 3.5mA | Entry settings  Group B  8- (a, b, c) PW: 60 125hz 3.5mA  Exit settings  Group A  8- (a, b, c) PW: 60 125hz 4.0mA | - Most ventral contacts superior - Improved right upper and lower limb dystonia. - Diaphragmatic dyskinesia improved significantly. - Mild improvement of lingual dystonia. - Increased STIM bilaterally |
| 4/12 | Entry settings  Group C  0- (a, b, c) PW: 60 125hz 3.4mA  Exit settings  Group D  0-(a, b, c) / 1- (a, b, c) PW 60 125Hz 0: 2.0mA, 1: 3.5mA  Group C  0- (a, b, c) PW: 90 125hz 3.0mA | Entry settings  Group C  8- (a, b, c) PW: 60 125hz 3.9mA  Exit settings  Group D  8- (a, b, c)/9 – (a, b, c) PW 60 125hz 8: 2.0m, 9: 3.5mA  Group C  8- (a, b, c) PW:90 125hz 3.0mA | - No additional dystonia improvement on high amplitudes. - More drooling and more parkinsonian. - Trial double monopolar 1^st^ and 2^nd^ best contact bilaterally Vs higher PW most ventral contact. |
| 5/12 | Entry settings  Group C  0- (a, b, c) PW: 90 125hz 3.0mA  Exit settings  Group B  0- (a, b, c) PW: 50 125hz 4.5mA  Group C  0- (a, b, c) PW: 120 125hz 1.8mA | Entry settings  Group C  8- (a, b, c) PW:90 125hz 3.0mA  Exit settings  Group B  8- (a, b, c) PW:50 125hz 4.5mA  Group C  8- (a, b, c) PW: 120 125hz 1.8mA | - Doble monopolar less effective overall. - New dystonic left shoulder pain. - Dysarthria worse - Trial 2 new groups low PW (50) and higher amplitude and higher PW (120) lower amplitude. |
| 6//12 | Entry settings  Group B  0- (a, b, c) PW: 50 125hz 4.5mA  Exit settings  Group B  0- (a, b, c) PW: 50 125hz 4.8mA  Group C  0- (a, b, c)/ 1- (a, b, c) PW 40 125Hz 0: 4.0mA 1: 1.9mA | Entry settings  Group B  8- (a, b, c) PW:50 125hz 4.5mA  Exit settings  Group B  8- (a, b, c) PW:50 125hz 4.8mA  Group C  8- (a, b, c)/ 9- (a, b, c) PW 40 125Hz 8: 4.0mA, 9: 1.9mA | - Group C (High PW) severe dysarthria, only tolerated for 3 days. - Group B overall marginally better, significant improvement in left shoulder dystonia. - Speech improved. - Mild dysphagia. - Increased STIM by 0.3mA B/L group B. - Trial of double monopolar levels 1 and 2 with lower PW. |
| 9/12 | Entry settings  Group C  0- (a, b, c)/ 1- (a, b, c) PW 40 125Hz 0: 4.0mA 1: 1.9mA  Exit settings  Group C  0- (a, b, c)/ 1- (a, b, c) PW 40 125Hz 0: 4.0mA 1: 1.9mA | Entry settings  Group C  8- (a, b, c)/ 9- (a, b, c) PW 40 125Hz 8: 4.0mA, 9: 1.9mA  Exit settings  Group C  8- (a, b, c)/ 9- (a, b, c) PW 40 125Hz 8: 4.0mA, 9: 1.9mA | - Double monopolar with lower PW marginally improved lingual dystonia and swallow. - Breathing/diaphragmatic dyskinesia ongoing but improved from prior to surgery. - Speech has not improved, unchanged with STIM OFF which triggers an immediate and distressing deterioration in facial and lingual dystonia. |
