# Supplementary material for: Clinical Outcomes with Prospective Brain Sensing Data Following Bilateral Globus Pallidus Deep Brain Stimulation in X‐Linked Dystonia Parkinsonism
Source: Mov Disord Clin Pract. 2025 Mar 15;12(7):1024–7. doi: 10.1002/mdc3.70044 (PMC12275003; doi:10.1002/mdc3.70044)
Supplement: Supplementary file 1 — Data S1. Reviewer comments and responses from authors. [file MDC3-12-1024-s002.docx]

**Supplementary Document 1: Reviewer comments and author responses**

**Although the reported results do not represent a major novelty, as they conclude that more data are needed to consider beta range hypersynchronisation as a biomarker for therapeutic response in DBS for patients with dystonia and XDP, I would consider this manuscript in letter format as a valuable contribution to the journal.

However, as presented, it seems to be a merely observational and subjective study, where little objective information can serve as a solid reference for readers. Regarding the brevity of the letter format, the authors should include as Supplementary Material more information about the Methods and Results:**

**Supplemental surgical approach: Describe the surgical technique, targeting strategies, implant coordinates, lead model, implant verification techniques, post-surgical MRI images or reconstructions as supplemental figures, etc.**

**Response:**

We acknowledge the limitations of our observations in these two subjects. The rarity of the condition (these are the only two patients in Ireland that have been diagnosed with DYT3) and the relative recency of sensing technology limits the number of patients we have to observe and the length of prospective follow up. We have changed the format of our manuscript to a letter as suggested.

A supplementary figure with MR imaging of lead implantation has been added (Supplementary figure 1).

**Surgical technique:**

All patients planned for surgery had a pre-operative CT and MRI scan under general anaesthetic within 6 weeks of surgery. CT and MRI were merged using the CT as the reference file. Stereotactic planning was performed on Brainlab (Munich, Germany) software. Targeting of the GPi was performed using Brainlab. Entry points were localised to a gyrus, avoiding sulci at entry and on the planned trajectory to the target nucleus. Planned trajectories were located as far from the ventricle as possible. Standard operative protocol was followed with an intra-operative CT with O-arm (Medtronic, United States) performed. The ROSA Robotic system (ROSA Brain, Zimmer Biomet, United States) was used for implantation of DBS leads. Medtronic sensight leads -33005 were used in both patients with Medtronic Percept Primary Cell implantable pulse generator (IPG). The intraoperative CT scan is fused using Brainlab software to the planning images. Fusion accuracy is verified by surgeon. The lead position is compared to the pre-operative target and the radial error is calculated. Routine operative protocol followed for tunnelling leads from scalp to chest incision. The leads are inserted into the IPG and secured with screws. The IPG is interrogated and impedances are recorded to ensure the device is working.

Lead co-ordinates:

| **Patient 1** | **Left Lead GPi** | **Right Lead Gpi** |
| --- | --- | --- |
| **Anterior to mid AC-PC point (mm)** | 3.73 | 2.72 |
| **Caudal to IC plane (mm)** | 4.16 | 5.47 |
| **Lateral to AC-PC line (mm)** | 21.93 | 20.86 |

| **Patient 2** | **Left Lead GPi** | **Right Lead Gpi** |
| --- | --- | --- |
| **Anterior to mid AC-PC point (mm)** | 2 | 4.04 |
| **Caudal to IC plane (mm)** | 2.33 | 5.35 |
| **Lateral to AC-PC line (mm)** | 22.15 | 18.27 |

**Supplemental analysis: Describe the IPG device data extraction (json files, etc.), filtering and processing tools, statistical analysis, and descriptions that help understand how the authors could draw objective conclusions beyond visual inspection of the limited plots offered by the simplified clinical programmer reports. Provide statistical proof of the significance of changes in the LFP profile (e.g., beta or sub-beta power attenuation)**

**Response:**

Raw data was extracted from the JSON files using custom MATLAB (Mathworks, Natick, MA USA. Version R2024b) scripts and was plotted using the MATLAB *plot* function. No additional post-processing was carried out on the data which is presented as downloaded from the Medtronic clinician programmer. LFT montage data file within JSON was plotted and visually inspected. The peak frequency detected was the peak noted in the JSON file and this was corroborated visually.

**Supplemental therapy description: Describe the stimulation settings, contacts, segments, stimulation parameters, and how they were adjusted throughout the prospective programming sessions. Please describe the BrainSense setup, Levels and Segments surveys, in-clinic stimulation titration, programming strategies and protocols throughout the study. A supplemental table or figure on this would be helpful.**

We followed our standard dystonia protocol for both patients based on the Toronto Western dystonia algorithm (1). This involved an initial monopolar review of all 4 contacts on each lead at standard settings (PW 60, 130 hz). On the basis of best therapeutic window and best anatomical placement focusing on more ventral contacts initially, stimulation was initiated at 2.5mA in paired contact groups beginning with the most ventral pair and for a period of 1 month per pair. Once best contact pair was selected, further programming iterations were made across multiple clinic visits, optimising pulse width and amplitude as tolerated with introduction of segmental stimulation where applicable. We detail these iterations in supplementary table 1 (Programming summary).

**Figure 1:
Please mark the beta (and sub-beta) peaks that the authors report in pat#1.
Describe whether the plots are from LFP Surveys recorded between levels or segments, which ones they are, or are they plots resulting from some processing, instead?**

**Response:**

The plots describe the LFPs in each hemisphere recorded in standard brainsense technique from level 1 and level 3 in both hemispheres. The LFP data was extracted from Medtronic JSONs and plotted in MATLAB, however, no additional processing was involved.

**In legends for pat#1 it reads B-1-3-6-18 m. In the manuscript it reads B–3–6–12 m. Please report consistently or explain assessment timelines.
Clinical scores show B-1-6-12 m for pat#1 and B-6-9 m for pat#2 (clinic reports in manuscript is 12 m). Please report consistently or explain the timelines for the assessments.**

Thank you for pointing out the inconsistency between the manuscript and legend. This has been rectified in the main body text for patient 1 LFP survey (line 73) and patient 2 timing of clinical assessments (line 85). With regard to the limited number of sensing surveys for patient 2, this was a consequence of the immediate discomfort caused during the survey while system was turned off, which became intolerable for the patient. When sensing surveys were run he would have acute bilateral painful facial and lingual dystonia. Similarly with clinical scales, there was some limitation to attended regular reviews in person and virtual appointments were offered, which did not allow 3 monthly clinical outcome scales.

**Line 102: The Discussion about the moderate response to DBS and lack of detection of clear peaks in beta or lower frequencies in pat#2 is interesting. Supported by the Supplementary material suggested about location of leads, and the proved benefit from surgery, I’d further explore the possibility of lead misplacements or just confirm the accurate implants as planned.**

**Response:**

We thank the reviewer for this comment. We indeed also found it interesting. Lead placement was confirmed to be satisfactory (see response above). Our interpretation is that this particular bulbar phenotype may not have the same LFP correlate as axial or appendicular phenotypes and may not respond as well to GPi DBS as is mentioned in the discussion. Although the patient with bulbar-predominant symptoms did not have a meaningful reduction in lingual dystonia, the acute deterioration in the stimulation OFF state, and relative stability over time, suggest that stimulation has had some positive impact on the natural course of the disease. Larger datasets of XDP patients with the full spectrum of phenotypes undergoing DBS are needed to further elaborate on our findings.

**In the supplementary table of Programming Summary, check for inconsistencies between Exit and Entry settings. If no changes were made between visits, values should match. Otherwise, explain.**

**Response:**
Thank you for identifying this. Where there is a change in amplitude between sessions, this was patient controlled amplitude increases. Both patients were given some freedom to adjust stimulation in the setting of dysarthria or other side effects and they did at times make unscheduled changes after phone consultations. I have added a line to explain this where it occurs.

**Looking at the table, group strategies according to the standard algorithm seem to apply only in pt#2**

**Response:**

While the Toronto Algorithm is very useful in initial programming and parameter adjustments, each centre has its own nuanced approach when it comes to programming based on a specific patient’s responses. This naturally was not a controlled trial of a particular stimulation approach so a standardised approach was not rigidly adhered to. We mention the Toronto protocol as a nod to the general principal we follow, all else being equal.

Patient one had acute and almost complete resolution of his axial and proximal lower limb dystonic spasms a the time of battery activation with stimulation on the level which he ultimately remained on. This response was not observed acutely with stimulation at any other level and low pyramidal side effect thresholds limited stimulation at level 1. Minor incremental adjustments of amplitude and pulse width were made to manage dysarthria. There was therefore no clinical rationale for exploration of alternative groups as per the Toronto protocol.

**It is still confusing to me about distinguishing between in-clinic visits and group trials programming according to the algorithm. I see in supp table for pt#1: BL-3M-6M-9M-12M-15M-18M, and for pt#2: BL-3/12-3/12-4/12-5/12-6/12-9/12.  Please clarify or reformat, and explain how this fits into the visit schedule reported in the main MS. (I suggest labelling the first column in the table, e.g. “Visit Schedule” or whatever)**

**Response:**

Both patients were programmed during COVID19 Pandemic and clinic shutdowns and utilisation of emergency clinic appointments were utilised to best care for these patients. Therefore, at times there was an inconsistent duration between appointments. Again, this was not a trial of DBS in Lubag rather a description of real-life clinical responses while taking the opportunity to make some novel, admittedly limited observations using new sensing technology in this rare disease. We also see patients, where possible, urgently if there has been acute decline in efficacy or distressing side effects. For that reason clinic visits were not always at standardised intervals. At times patient 2 did not wish to have sensing survey run as it involves a brief time off STIM, which was intolerable.

**Format parameters and report them throughout the table ALWAYS in the same order (e.g. PW, Frequency, Amplitude). Detail missing group label in pt#2 3/12**

**Response:**

Thanks you for pointing out these discrepancies, they have been corrected in the manuscript.

**Please check for each visit: Exit settings should be preceded by Entry settings for the involved group, otherwise clarify in table legend that the mentioned group was set on a previous visit (where it should be detailed).

Please, also check BL values for pt#2 according to the initial programming described based on the Toronto algorithm.**

**Response:**

BL STIM parameters for patient limited to 2 groups based on therapeutic windows. Where exit and entry setting differ, there has been an increase in STIM by patient. Any discrepancy between exit and entry setting has been clarified in clinical assessment column.

**Medtronic devices report misleading values when stimulating in segmented mode. Total amplitude shown is calculated as if all contacts where recruited, however the actual current delivered corresponds to the sum of individual contacts or segments, which may lead to the misconception that stimulation was increased when it was actually decreased, just like pt#1 (GPiR, Exit settings in 3M); please write a line in supp material explaining this issue as all directional settings may lead to misinterpreted actual stimulation intensities**

In the interest of making it STIM settings as clear as possible, I have removed the figure for total STIM, which we agree can be misleading and just included amplitude at individual segments.

References:

1.	Picillo M, Lozano AM, Kou N, Munhoz RP, Fasano A. Programming Deep Brain Stimulation for Tremor and Dystonia: The Toronto Western Hospital Algorithms. Brain Stimul. 2016;9(3):438-52.
